# Individual courses and determinants of fear of cancer recurrence in long-term breast cancer survivors with and without recurrence

**DOI:** 10.1007/s00520-021-06329-z

**Published:** 2021-06-17

**Authors:** Paula Heidkamp, Clara Breidenbach, Kati Hiltrop, Christoph Kowalski, Anna Enders, Holger Pfaff, Birgitta Weltermann, Franziska Geiser, Nicole Ernstmann

**Affiliations:** 1 Center for Health Communication and Health Services Research (CHSR), Department for Psychosomatic Medicine and Psychotherapy, University Hospital Bonn, Bonn, Germany; 2 Center for Integrated Oncology Bonn (CIO), University Hospital Bonn, Bonn, Germany; 3 German Cancer Society, Berlin, Germany; 4 Federal Centre for Health Education, Cologne, Germany; 5 Institute for Medical Sociology, Health Services Research and Rehabilitation Science (IMVR), University of Cologne, Cologne, Germany; 6 Institute of General Practice and Family Medicine, University of Bonn, Bonn, Germany; 7 Department of Psychosomatic Medicine and Psychotherapy, University Hospital Bonn, Bonn, Germany

**Keywords:** Anxiety, Breast cancer, Fear of cancer recurrence, Oncology, Relapse, Survivorship

## Abstract

**Objective:**

This study investigated the prevalence, individual courses, and determinants of fear of cancer recurrence (FoR) in long-term breast cancer survivors (BCSs) with and without recurrence.

**Methods:**

A total of 184 breast cancer survivors were surveyed at four measurement time points: during hospitalization (T1), 10 weeks (T2), 40 weeks (T3), and 5–6 years (T4) after hospital discharge. Descriptive statistics, chi-square tests, and logistic regression were performed.

**Results:**

Respondents were females and 57 years old, on average. At T1, T3, and T4, 54.8%, 31.6%, and 29.7% of BCSs, respectively, were classified as having dysfunctional levels of FoR. Dysfunctional FoR decreased from T1 to T3 (χ^2^(1) = 17.11, p = 0.000; N = 163) and remained stable afterwards. Eight subgroups of individual courses of FoR over time could be described: (1) constant functional FoR; (2) constant dysfunctional FoR; (3) improving from dysfunctional to functional FoR from T1 to T3; (4) improving from dysfunctional to functional FoR from T3 to T4; (5) worsening from functional to dysfunctional FoR from T1 to T3; (6) worsening from functional to dysfunctional FoR from T3 to T4; (7) dysfunctional FoR at T1 and T4, and functional FoR in between; and (8) functional FoR at T1 and T4, and dysfunctional FoR in between. Logistic regression analysis revealed that being divorced/widowed, showing high levels of fatigue, being treated by chemotherapy, and having low confidence in treatment were associated with dysfunctional FoR 5 to 6 years after diagnosis (Nagelkerkes’ Pseudo-R2 = 0.648).

**Conclusions:**

The findings reveal that FoR is a significant issue in long-term BCSs and has the potential to become a persistent psychological strain. We emphasize the need for increased awareness of FoR among BCSs and the need for support programs.

## Background

Even years after diagnosis and active treatment, cancer survivors suffer from their disease in multiple ways and report lower levels of quality of life compared to the non-affected population [1, 2]. A widespread source of psychological distress, which is not only one of the most important strains in cancer patients in acute treatment [3], but also affects long-term cancer survivors, is the fear of cancer recurrence (FoR) [4, 5]. FoR is defined as “Fear, worry, or concern relating to the possibility that cancer will come back or progress.” [6] FoR is basically described as an appropriate reaction to cancer and its life-threatening potential and can enhance motivation, for example, to keep appointments for follow-up care or to engage in a healthy lifestyle [7]. However, FoR can also become dysfunctional when clinically significant severity is reached [6–8] and can be associated with lower quality of life, depression, and anxiety, even years after diagnosis [9, 10]. There is an expert consensus that dysfunctional FoR comprises certain characteristics, such as high levels of preoccupation and worry, which are persistent, as well as hypervigilance to bodily symptoms [6, 8]. In addition, high levels of FoR are associated with a higher risk of diagnosis with a psychiatric disorder compared to non-clinical levels [11]. Even years after diagnosis, a substantial number of cancer survivors suffer from FoR, which can be classified as dysfunctional [9–11]. However, studies that use predefined cutoff values for the clinical significance of FoR are rare; therefore, interpretations in terms of functional or dysfunctional levels of FoR in cancer survivors are limited [4, 5]. Regarding the course of FoR in cancer survivors over time, most studies found FoR to be stable [4, 5]. However, for a better understanding of FoR in long-term cancer survivors, not only mean values but also more individual courses of FoR need to be considered. The first studies that investigated group-based trajectories of FoR in cancer patients identified three patterns: constant low FoR over time, constant high FoR over time, and decreasing FoR over time [12, 13]. As these studies focused on the first year of the cancer, generalization to long-term cancer survivors is limited. Furthermore, for a better understanding of individual experiences of FoR in the long term, it should be considered whether breast cancer survivors (BCSs) actually have a recurrence over the course of the disease or not. Previous studies found both positive associations between having a recurrence and FoR and no significant relationship between these variables [5, 9].

Therefore, the present study aims to (1) investigate the prevalence of functional and dysfunctional FoR in long-term BCSs over a period of 5 to 6 years after diagnosis, (2) describe individual courses of functional and dysfunctional levels of FoR of BCSs with and without recurrence from hospitalization to 5–6 years after diagnosis, and (3) analyze the association of dysfunctional FoR in long-term BCSs 5–6 years after diagnosis with sociodemographic and health- and treatment-related variables.

## Methods

### Study design and participants

**Fig. 1:**
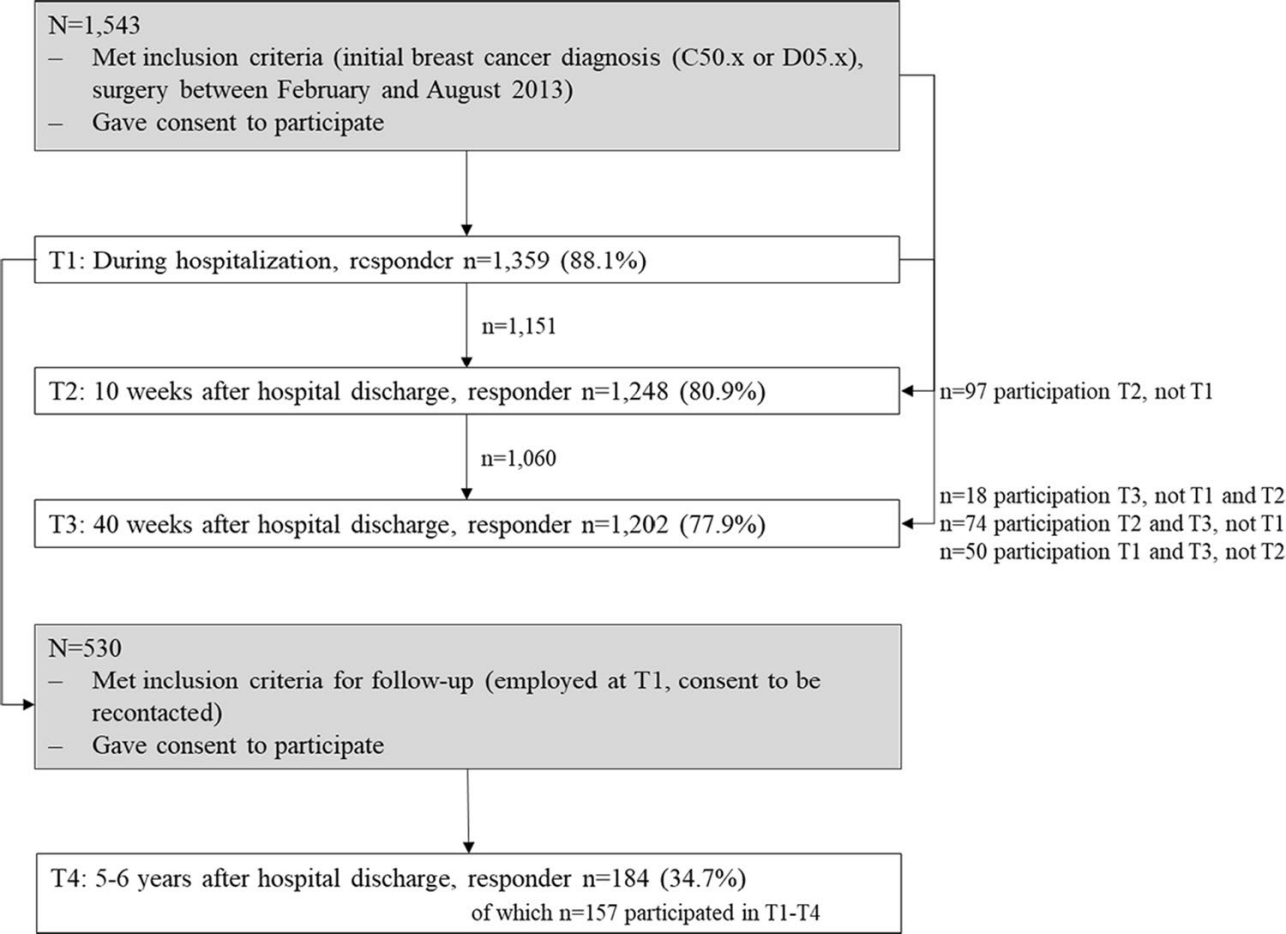
Flow of participants. Note: The number of respondents composes of participants who consecutively participated in every survey wave and those who participated at least once. Dropouts occurred due to nonresponse, death, or unverifiable addresses

The B-CARE project (“breast cancer patients’ return to work”) was initiated in 2018 to study sociodemographic and psychosocial determinants of breast cancer patients’ use of medical rehabilitation and return to work. B-CARE is a longitudinal study that uses survey data of breast cancer patients from four measurement time points: during hospitalization (T1), 10 weeks after hospital discharge (T2), 40 weeks after hospital discharge (T3), and 5–6 years after hospital discharge (T4) (T1: n = 1359; T2: n = 1248; T3: n = 1202; T4: n = 184). The flow of participants is shown in Fig. 1. Data from the first three measurement time points were collected during the preceding PIAT project (“Strengthening patient competence: Breast cancer patients’ information and training needs”). The PIAT study was initiated in 2013 and patients with an initial diagnosis of breast cancer (n = 1359; C50.x or D05.x) from n = 60 breast cancer centers throughout Germany were recruited for the study [14, 15]. Patients were surveyed during hospitalization. They received a questionnaire via mail 10 weeks and 40 weeks after hospital discharge. In 2019, B-CARE carried out a follow-up survey of a subsample of 530 patients who gave their consent to be re-contacted and who were working at the time of their diagnosis. Hundred and eighty-four patients participated in the survey 5 to 6 years after hospital discharge (response rate = 35%). Responder and non-responder at T4 did not differ significantly regarding FoR, medical, psychosocial, and sociodemographic characteristics (e.g., UICC TNM stage, number of comorbidities, age) (analyses not shown). Detailed information on the study design and sampling process can be found elsewhere [16].

### Measurements

#### Fear of cancer recurrence

The short form of the *Fear of Progression Questionnaire* (FoP-Q-SF) [17] was used to collect data at T1, T3, and T4. The FoP-Q-SF consists of 12 items and includes four subscales (affective reactions, partnership/family, occupation, and loss of autonomy) of the original version [18]. Table 1 gives an overview of the items of the FoP-Q-SF [19]. The 12 items were assessed using a 5-point Likert scale (1 = “never” to 5 = “very often”), leading to total scores ranging from 12 to 60, where higher values indicate higher levels of FoR. A cutoff score of 34 or above was used to identify dysfunctional levels of FoR [20]. At T3, item 12 of the FoP-Q-SF was missing in the survey, leading to a total of 11 items. In order to ensure that the results (possible range: 11–54) were still comparable to the results of the original 12-item version, the total scores were standardized to the original metric (possible range: 12–60). Therefore, total scores of the 11 items were divided by their possible maximum values based on the respective number of missing values for each participant. Values were then multiplied by the maximum possible value of the original instrument.

**Table 1:** Items of the short form of the Fear of Progression Questionnaire (FoP-Q-SF)

(1) Being afraid of disease progression
(2) Being nervous prior to doctor’s appointment or periodic examinations
(3) Being afraid of pain
(4) Being afraid of becoming less productive at work
(5) Having physical sensations, e.g., rapid heartbeat, stomach ache, nervousness
(6) Being afraid of the possibility that the children could contract cancer
(7) Being afraid of relying on strangers for activities of daily living
(8) Being afraid of no longer be able to pursue hobbies
(9) Being afraid of severe medical treatments in the course of the illness
(10) Worrying that medication could damage the body
(11) Worrying about what will become of the family
12) Being afraid of not being able to work anymore

#### Determinants of FoR

To identify the determinants of functional and dysfunctional FoR at T4, data on sociodemographic and health- and treatment-related variables were collected. Table 2 gives an overview of the variables measured at T1, T2, T3, and T4. Sociodemographic data, such as age and number of children, were assessed in the questionnaire at T4. The variables family status and employment status were assessed at T1, T2, T3, and T4. In order to analyze the most current data on family status and employment status, these variables were only examined at T4. The variable “highest vocational education level achieved” was assessed at T1. Data on cancer classification were added by the clinical personnel at T1, according to the categories of the Union Internationale Contre le Cancer (UICC) [21]. The number of comorbidities was assessed at T1 and T4. In order to use the most current data on comorbidities, the variable was only examined at T4. Recurrence status was assessed using the questionnaires at T4. To collect data on fatigue, the *Fatigue Assessment Questionnaire* [22] was used at T4. The FAQ consists of 20 items and three subscales (physical, affective, and cognitive fatigue). Treatment-related characteristics, such as being treated by chemotherapy, radiation therapy, or hormonotherapy, were assessed in the questionnaire at T1, T2, and T3. If a treatment type was provided at least once over the three measurement time points, it was considered given. The single item variable “confidence in treatment,” which measures a positive belief in the outcome of the treatment, was assessed in the questionnaire at T1 using a 10-point Likert scale (0 = “not confident”, 10 = “confident”). The 20 items were assessed using a four-point Likert scale (0 = “not at all” to 3 = “very much”), leading to the highest possible sum score of 60, where higher values indicate higher levels of fatigue.

**Table 2:** Variables measured at T1, T2, T3, and T4

Variables	T1	T2	T3	T4
Fear of cancer recurrence (FoP-Q-SF)	x		x	**x**
Sociodemographic variables				
Age				**x**
Family status	x	x	x	**x**
Number of children				**x**
Employment status	x	x	x	**x**
Vocational education level	**x**			
Health-related variables				
Cancer classification (UICC)	**x**			
Number of comorbidities	x			**x**
Recurrence status				**x**
Fatigue (Fatigue Assessment Questionnaire)				**x**
Treatment-related variables				
Chemotherapy	**x**	**x**	**x**	
Radiation therapy	**x**	**x**	**x**	
Hormonotherapy	**x**	**x**	**x**	
Confidence in treatment	**x**			

Note: Variables examined in the logistic regression analysis in bold

### Statistical methods

Statistical analyses were conducted using IBM SPSS Statistics version 24. Missing values were deleted list wise. To describe the prevalence of functional and dysfunctional levels of FoR over time, descriptive statistics and chi-square tests were conducted.

Individual courses of FoR were described for each participant with data on FoR available at T1, T3, and T4. Furthermore, individual courses of FoR were illustrated separately for participants with and without recurrence. By using the cutoff of 34, the participants were classified as having either functional or dysfunctional levels of FoR at T1, T3, and T4. Based on theoretical assumptions regarding the number of possible courses (3 time points, 2 values), eight different groups of courses were expected: (1) functional FoR at T1, T3, and T4; (2) dysfunctional FoR at T1, T3, and T4; (3) functional FoR at T1 and T3, and dysfunctional FoR at T4; (4) functional FoR at T1 and dysfunctional FoR at T3, and T4; (5) dysfunctional FoR at T1 and T3, and functional FoR at T4; (6) dysfunctional FoR at T1 and functional FoR at T3, and T4; (7) functional FoR at T1 and T4, and dysfunctional FoR at T3; (8) dysfunctional FoR at T1 and T4, and functional FoR at T3. Depending on the level of FoR at each time point, participants were assigned to a subgroup.

To investigate the determinants of functional or dysfunctional FoR at T4, logistic regression modeling, which facilitated the estimation of the sociodemographic and health- and treatment-related characteristics as predictors of FoR with the help of the maximum likelihood method, was applied. The variables were included in a block-wise manner. The first model (M1) contains sociodemographic, health-related, and treatment-related variables. In the second model, the variables fatigue and confidence in treatment were added.

## Results

### Descriptive results

The sample consisted of 184 female breast cancer survivors. N = 145 reported no cancer recurrence and n = 36 reported to have had a recurrence. The sample characteristics are reported in Table 3. Data on FoR at T1, T3, and T4 was available for n = 155 participants. Of those, n = 122 reported no cancer recurrence and n = 32 reported to have had a recurrence. For n = 1, there was no data on recurrence status available.

**Table 3:** Characteristics of study participants (n = 184)

		Abs (%)	Mean	Standard deviation	Min–max
Dependent variable: fear of recurrence (FoR)					
T1			35.10	8.61	18–60
	Missing	13 (7.1)			
T3			31.23	8.46	16.36–54.55
	Missing	20 (10.9)			
T4			29.14	9.06	12–54
	Missing	8 (4.3)			
Independent variables: sociodemographic variables					
Age in years (T4)			56.93	6.82	36–79
	Missing	15 (8.2)			
Marital status (T4)	Married	134 (72.8)			
	Single	17 (9.2)			
	Divorced/widowed	33 (17.9)			
	Missing	0 (0.0)			
Number of children (T4)			1.52	0.98	0–4
	Missing	0 (0.0)			
Vocational training (T1)	Low	106 (57.6)			
	Intermediate	34 (18.5)			
	High	33 (17.9)			
	Missing	11 (6.0)			
Employment status (T4)	Full time	51 (27.7)			
	Part time/occupational rehabilitation	85 (46.2)			
	Non-working	42 (22.8)			
	Missing	6 (3.3)			
Independent variables: health-related variables					
UICC TNM stage (T1)	UICC 0/1	79 (42.9)			
	UICC 2/3/4	71 (38.6)			
	Missing	34 (18.5)			
Number of comorbidities (T4)			1.01	1.10	0–5
	Missing	17 (9.2)			
Recurrence (T4)	No	145 (78.8)			
	Yes	36 (19.6)			
	Missing	3 (1.6)			
Fatigue (T4)			20.75	15.62	0–59
	Missing	1 (0.5)			
Independent variables: treatment-related variables					
Chemotherapy (T1, T2, and T3)	No	95 (51.6)			
	Yes	80 (43.5)			
	Missing	9 (4.9)			
Radiation therapy (T1, T2, and T3)	No	113 (61.4)			
	Yes	62 (33.7)			
	Missing	9 (4.9)			
Hormonotherapy (T1, T2, and T3)	No	54 (29.3)			
	Yes	121 (65.8)			
	Missing	9 (4.9)			
Confidence in treatment (T1)			8.99	1.29	2–10
	Missing	11 (6.0)			

**Fig. 2:**
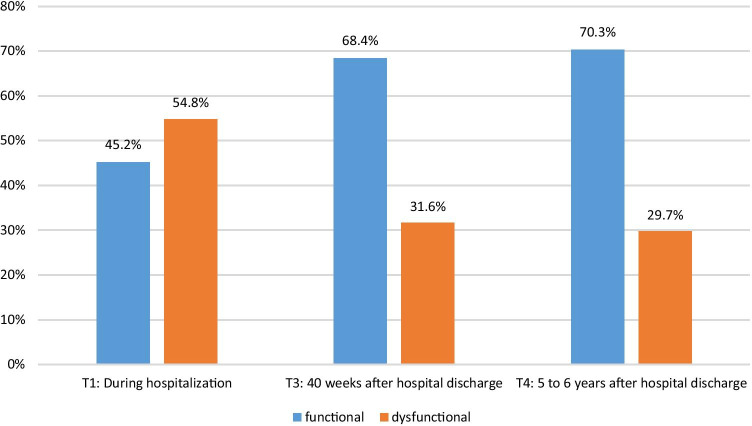
Levels of fear of recurrence at T1, T3, and T4. N = 155

Figure 2 shows the proportions of functional and dysfunctional FoRs over time. The results revealed a decline in dysfunctional levels of FoR from T1 to T3 and a stable course afterwards.

**Fig. 3:**
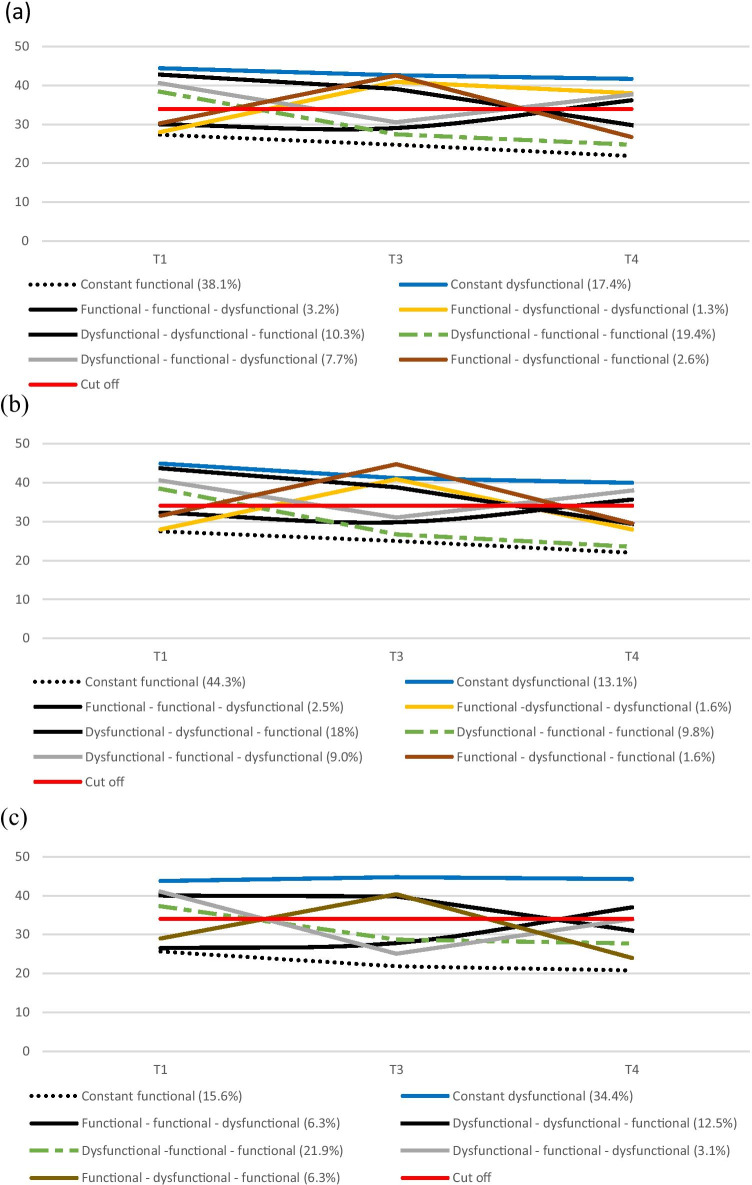
(**a**) Individual courses of fear of cancer recurrence from T1 to T4 (n = 155); (**b**) individual courses of fear of cancer recurrence from T1 to T4 of BCSs without recurrence (n = 122); (**c**) individual courses of fear of cancer recurrence from T1 to T4 of BCSs with recurrence (n = 32)

Regarding individual courses of FoR over time, eight subgroups could be identified (Fig. 3a): (1) 38.1% of BCSs reported constant functional levels of FoR at all measurement time points; (2) 17.4% of BCSs showed constant dysfunctional levels of FoR at all measurement time points; (3) 19.4% of BCSs improved from a dysfunctional to a functional level of FoR from T1 to T3 and remained functional afterwards; (4) 10.3% of BCSs reported dysfunctional levels of FoR at T1 and T3 and improved to a functional level at T4; (5) 1.3% had dysfunctional levels of FoR from T1 to T3 and remained dysfunctional afterwards; (6) 3.2% showed functional levels of FoR at T1 and T3 and dysfunctional FoR at T4; (7) 7.7% of BCSs showed dysfunctional levels of FoR at T1 and T4, but reported functional FoR at T3; and (8) 2.6% of BCSs showed functional levels of FoR at T1 and T4, but reported dysfunctional FoR at T3.

With regard to BCSs without recurrence (Fig. 3b), (1) 44.3% of BCSs reported constant functional levels of FoR; (2) 13.1% of BCSs showed constant dysfunctional levels of FoR; (3) 18% of BCSs improved from a dysfunctional to a functional level of FoR from T1 to T3 and remained functional afterwards; (4) 9.8% of BCSs reported dysfunctional levels of FoR at T1 and T3 and improved to a functional level at T4; (5) 1.6% had dysfunctional levels of FoR from T1 to T3 and remained dysfunctional afterwards; (6) 2.5% showed functional levels of FoR at T1 and T3 and dysfunctional FoR at T4; (7) 9.0% of BCSs showed dysfunctional levels of FoR at T1 and T4, but reported functional FoR at T3; and (8) 1.6% of BCSs showed functional levels of FoR at T1 and T4, but reported dysfunctional FoR at T3.

Regarding BCSs with recurrence (Fig. 3c), seven subgroups could be identified: (1) 15.6% of BCSs reported constant functional levels of FoR; (2) 34.4% of BCSs showed constant dysfunctional levels of FoR; (3) 21.9% of BCSs improved from a dysfunctional to a functional level of FoR from T1 to T3 and remained functional afterwards; (4) 12.5% of BCSs reported dysfunctional levels of FoR at T1 and T3 and improved to a functional level at T4; (5) 6.3% of BCSs showed functional levels of FoR at T1 and T3 and dysfunctional FoR at T4; (6) 3.1% of BCSs showed dysfunctional levels of FoR at T1 and T4, but reported functional FoR at T3; and (7) 6.3% of BCSs showed functional levels of FoR at T1 and T4, but reported dysfunctional FoR at T3.

### Multivariate results

Chi-square tests of independence were performed to examine the relationship between functional and dysfunctional FoR and time. There was a significant relationship between the levels of FoR and time for T1 and T3 (χ^2^(1) = 17.11, p = 0.000; N = 163). Dysfunctional levels of FoR were more likely at T1 than at T3, indicating a decrease in dysfunction and an increase in functional FoR over time. There was no significant association between functional or dysfunctional levels of FoR and time at T3 and T4 (χ^2^(1) = 0.14, *p* = 0.71; N = 156).

To analyze the determinants of dysfunctional FoR 5 to 6 years after initial diagnosis, a logistic regression model was estimated. Table 4 shows the results of the logistic regression for FoR at T4.

**Table 4:** Logistic regression model with fear of recurrence as the dependent variable (n = 140)

		Model 1		Model 2	
Variables	Response trait	OR	95% CI	OR	95% CI
Age in years	Metric	0.90*	0.82–1.00	0.98	0.87–1.10
Marital status	Married	0.12**	0.03–0.53	0.14*	0.02–0.83
	Single	0.17	0.20–1.43	0.57	0.04–9.04
	Divorced/widowed	1.0		1.0	
Number of children	Metric	1.17	0.70–1.96	1.01	0.56–1.81
Vocational training	Low	2.02	0.59–6.99	1.77	0.39–8.08
	Intermediate	0.76	0.16–3.72	0.59	0.90–3.86
	High	1.0		1.0	
Employment status	Full time	0.78	0.16–3.99	2.06	0.24–17.43
	Part time/occupational rehabilitation	0.85	0.21–3.56	2.26	0.40–12.69
	Non-working	1.0		1.0	
UICC TNM stage	Stage 0/I	0.75	0.23–2.38	0.76	0.19–3.12
	Stage II/III/IV	1.0		1.0	
Number of comorbidities	Metric	2.46**	1.49–4.04	1.37	0.75–2.52
Recurrence	No	2.23	0.71–6.94	1.50	0.38–5.98
	Yes	1.0			
Chemotherapy	Yes	10.48*	2.71–40.53	5.53*	1.22–25.15
	No	1.0		1.0	
Radiation therapy	Yes	2.63	0.81–8.59	2.99	0.74–12.12
	No	1.0		1.0	
Hormonotherapy	Yes	2.95	0.92–9.46	2.55	0.67–9.72
	No	1.0		1.0	
Fatigue	Metric			1.11**	1.05–1.17
Confidence in treatment	Metric			0.63*	0.41–0.97
Nagelkerkes-R^2^		0.461		0.632	

*p < 0.05; **p < 0.01. Functional FoR = 0, dysfunctional FoR = 1

Model 1 shows that older adults (OR = 0.90; 95% CI = 0.82–1.00) and those who reported more comorbidities (OR = 2.46; 95% CI = 1.49–4.04) were more likely to report dysfunctional FoR at T4. BCSs who were married were less likely to report dysfunctional FoR (OR = 0.12; 95% CI = 0.03–0.53) than those who were divorced or widowed. Being treated with chemotherapy (OR = 10.48; 95% CI = 2.71–40.53) was associated with a higher risk for dysfunctional FoR at T4.

After inclusion of the variables fatigue and confidence in treatment (model 2), the association between age and FoR (OR = 0.98; 95% CI = 0.87–1.10) and that between comorbidities and FoR (OR = 1.37; 95% CI = 0.75–2.52) was no longer significant. Like Model 1, Model 2 shows that married BCSs were less likely to report dysfunctional FoR 5 to 6 years after hospital discharge (OR = 0.14; 95% CI = 0.02–0.83) than those who were divorced or widowed. Being treated with chemotherapy (OR = 5.53; 95% CI = 1.22–25.15) was associated with a higher risk for dysfunctional FoR at T4. BCSs who reported lower confidence in treatment at T1 (OR = 0.63; 95% CI = 0.41–0.97) and higher levels of fatigue (OR = 1.11; 95% CI = 1.05–1.12) were more likely to show dysfunctional levels of FoR at T4.

## Discussion

Regarding the prevalence of FoR among BCSs, the results show that 5 to 6 years after hospitalization, most BCSs (70%) experienced functional levels of FoR. However, almost one-third of the BCSs reported dysfunctional levels of FoR. This number is higher compared to that reported in other studies [9, 10]. These differences may be explained by the younger age and employment status of the participants in the study sample. There is strong evidence that younger age is associated with higher FoR [5, 23]. With regard to employment status, it must be noted that the FoP-Q-SF includes two items that evaluate occupational worries, leading to a higher FoR score if working life is still an issue.

Regarding the average course of FoR over time, the results show a decrease in dysfunctional and an increase in functional levels of FoR during the first 40 weeks after hospital discharge and a stable course 5 to 6 years after hospital discharge. These results are comparable to those of other studies that show a decrease in FoR during the first year after diagnosis but no long-term effect of time after diagnosis [9, 24–26].

Considering the overall sample, most BCSs showed constant functional levels of FoR (38.1%), followed by BCSs who improved from a dysfunctional to a functional level over time (29.7%) and BCSs who reported constant dysfunctional FoR (17.4%). Only a small number of BCSs showed a functional level of FoR at T1 and a dysfunctional level of FoR at T4 (4.5%). As the majority of BCSs in the present study showed steady FoR in terms of functional or dysfunctional levels, our findings partly support those of studies that found FoR in cancer survivors to be stable over time [4, 5]. On the other hand, in almost 45% of the participants, the intensity of FoR changed over the course of 5 to 6 years, indicating that time has the potential to affect FoR.

When comparing the individual courses of FoR of BCSs with and without recurrence, it appears that BCSs with recurrence descriptively show more often constant dysfunctional FoR (34.4%) and less often constant functional levels of FoR (15.6%) over the course of 5–6 years after diagnosis than BCSs without recurrence. These results suggest a positive association between having had a recurrence and dysfunctional FoR and are in accordance with previous research [9]. Furthermore, the results suggest that BCSs with recurrence show more dysfunctional FoR right from the start, even before having the actual recurrence.

The results of the logistic regression revealed a significant association between the marital status and the intensity of FoR in long-term BCSs. Women who were married were less likely to report dysfunctional FoR 5 to 6 years after diagnosis than women who were divorced or widowed. However, the effect was small (OR = 0.14). This association could be explained in terms of social support, which is probably more available for married than for divorced or widowed BCSs. Social support acts as a protective factor to lower the impact of stressors [27] and is negatively associated with higher FoR in long-term cancer survivors [28].

Furthermore, BCSs who were suffering from higher levels of fatigue were more likely to show dysfunctional FoR 5 to 6 years after diagnosis than BCSs who experience low levels of fatigue. These results are in accordance with previous research, which found strong evidence for the association between fatigue and other physical symptoms related to higher FoR [5]. This relationship might be explained by the ability of bodily sensations to trigger thoughts about cancer recurrence and the corresponding emotions and the fact that hypervigilance to bodily symptoms is a key characteristic of dysfunctional FoR [8].

BCSs who were treated with chemotherapy were at a higher risk for dysfunctional FoR at T4. These results are comparable to those of other studies [10, 29]. Adjuvant therapy is performed in order to reduce the risk of cancer recurrence and to draw the patients’ attention to this risk, leading to higher levels of FoR. Another explanation for this association might be the long-term side effects of the therapy, which still cause physical symptoms years after treatment. Furthermore, treatment with chemotherapy could be an indicator of the severity of the cancer, leading to higher levels of FoR. However, cancer staging was not a significant predictor of FoR in the present study. In addition, chemotherapy often is recommended to younger cancer patients, who tend to show higher levels of FoR [5, 23].

Moreover, BCSs who reported lower confidence in treatment during hospitalization were more likely to show dysfunctional levels of FoR at T4 compared to BCSs who were more optimistic about their therapy. These results confirm those of previous studies that identified pessimism as a risk factor for cancer-related health concerns, anxiety, and depression in cancer survivors [30, 31].

The first model revealed significant associations between age and FoR at T4 as well as comorbidities and FoR at T4. After including the variables fatigue and confidence in treatment, the associations were no longer significant. This effect might be explained by the correlations between the variables, leading to the disappearance of the associations in the second model.

### Study limitations

There are some study limitations which should be considered when interpreting the presented results.

The B-CARE study is an observational, and not an experimental, study. Therefore, only associations, but not causality, can be drawn from the results. The present study used a longitudinal study design with data collection at several measurement time points over a time span of 5 to 6 years. It is possible that study participants differ from non-participants in terms of health condition and emotional strain, which could have affected their motivation or ability to participate in the study. This bias could have led to an underestimation of the FoR of the BCSs. However, responder and non-responder at T4 did not differ significantly regarding FoR, medical, psychosocial, and sociodemographic characteristics.

Moreover, the use of a written survey could have resulted in the exclusion of patients who do not have sufficient reading, writing, or language skills. In terms of generalizability, it should be noted that only BCSs who worked prior to their diagnosis were considered in the present study. Therefore, a bias toward a younger and more educated sample is possible.

By interpreting the individual courses of FoR, it is important to consider that the courses were illustrated descriptively and that some of the subgroups have low sample sizes.

### Clinical implications

The presented findings emphasize the relevance of FoR in BCSs and indicate that a significant number of BCSs suffer from dysfunctional fear and worries even years after diagnosis. As different courses of FoR have been illustrated, continuous screening for FoR over the course of the disease and survivorship is required. Therefore, health personnel in inpatient and outpatient settings should be aware of FoR and its characteristics, which indicate dysfunctional levels of FoR (e.g., hypervigilance to bodily symptoms). The presented findings on the determinants of dysfunctional FoR in long-term BCSs could be helpful in identifying high-risk groups, such as those who are divorced or widowed, those who have been treated using chemotherapy, those who report low confidence in treatment right from the start, and those who report high levels of fatigue as a long-term consequence of the cancer. In addition, therapeutic interventions could be derived from the reported risk factors, for example, activation of social networks or cognitive restructuring regarding the meaning of bodily symptoms. At the same time, it should be acknowledged that there are many proven interventions for FoR (e.g., ConquerFear [32]). The study results reveal that a substantial number of BCSs have constant dysfunctional levels of FoR over a period of 5 to 6 years, indicating that for many BCSs, time, per se, does not have a curative effect. Therefore, more support options accessible for both cancer patients and long-term cancer survivors in the health care system are required.

## Conclusion

Overall, the findings from this study suggest that FoR is a significant issue among long-term BCSs. Almost one-third of the BCSs reported dysfunctional levels of FoR 5 to 6 years after diagnosis, indicating the potential of FoR to be a serious and persistent psychological strain following cancer. The findings support the need for increased awareness of the presence of FoR during and years after treatment and the need for support programs. Attention should be given to those who are divorced or widowed, who have undergone chemotherapy, who show low confidence in treatment, and who report high levels of fatigue. To gain a deeper understanding of FoR in cancer survivors, further studies involving both quantitative and qualitative data are needed.

## Data Availability

Research data are not shared.
